# Comparative Analysis of [Au(en)_2_]^3+^ and [Pt(en)_2_]^2+^ non Covalent Binding to Calf Thymus DNA

**DOI:** 10.1155/MBD.2000.253

**Published:** 2000

**Authors:** Giordana Marcon, Tim O'Connell, Pierluigi Orioli, Luigi Messori

**Affiliations:** 1 CIRCMSB Unit of Florence via Gino Capponi 7 Florence I-50121 Italy; 2 Department of Chemistry University of Florence Florence I-50121 Italy

## Abstract

Reactions of the complexes bisethylendiammine gold(III) and bisethylendiammine platinum(II) with calfthymus DNA were comparatively analysed. Both complexes bind DNA non-covalently most probably on the basis of electrostatic interactions. Binding of either complex at low ratios results into modest modifications of B-type DNA conformations, as detected by CD. Far larger CD alterations are observed at high ratios. The gold(III) chromophore is scarcely perturbed by DNA addition Binding of [Au(en)_2_]Cl_3_ to calf thymus DNA is reversed by sodium cyanide. By analogy with the case of [Pt(en)_2_]Cl_2_ it is suggested that Auen acts as a minor groove binder.


### COMPARATIVE ANALYSIS OF $[Au(en)_2]^{3+}$ AND $[Pt(en)_2]^{2+}$ NON COVALENT BINDING TO CALF THYMUS DNA

Giordana Marcon[1], Tim O'Connell[2], Pierluigi Orioli[2] and Luigi Messori*[2]

[1] CIRCMSB, Unit of Florence, via Gino Capponi 7, I-50121 Florence, Italy
[2] Department of Chemistry, University of Florence, I-50121 Florence, Italy
<messori@cerm.unifi.it>

### ABSTRACT

Reactions of the complexes bisethylendiammine gold(III) and bisethylendiammine platinum(II) with calf thymus DNA were comparatively analysed. Both complexes bind DNA non-covalently most probably on the basis of electrostatic interactions. Binding of either complex at low ratios results into modest modifications of B-type DNA conformations, as detected by CD. Far larger CD alterations are observed at high ratios. The gold(III) chromophore is scarcely perturbed by DNA addition Binding of $[Au(en)_2]Cl_3$ to calf thymus DNA is reversed by sodium cyanide. By analogy with the case of $[Pt(en)_2]Cl_2$ it is suggested that Auen acts as a minor groove binder.

### INTRODUCTION

There is considerable interest in evaluating non-covalent interactions of transition metal complexes to DNA since the detailed knowledge of these interactions may contribute to elucidate the mechanisms of metal-DNA recognition [1,2]. For example extensive work has been carried out on binding of the cations hexammine cobalt(III) and tris ethylendiamine cobalt(III) to DNA; both complexes were shown to be very effective in recognizing specific DNA sequences and in modifying DNA conformation through formation of electrostatic and hydrogen bond interactions [3-5]. The non-covalent interactions of several ruthenium complexes with DNA and with synthetic oligonucleotides have been investigated in detail by Barton and by other groups [6-8]. Recently, Collins et al reported on the reaction of $[Pt(en)_2]Cl_2$ with the DNA dodecamer $d(CAATCCGGATTG)_2$ and demonstrated, by high resolution $^1H$ NMR spectroscopy, that this complex binds non-covalently the oligonucleotide in the minor groove at AT rich regions [9].

In the last years we have been working on the development, the characterization and the pharmacological evaluation of gold(III) complexes as potential antitumor agents [10-12]; among the investigated compounds, the cationic square planar bisethylendiamine gold(III) complex ($[Au(en)_2]Cl_3$ hereafter; scheme I) showed favorable cytotoxic properties toward the A2780 human ovarian cancer cell line [12].

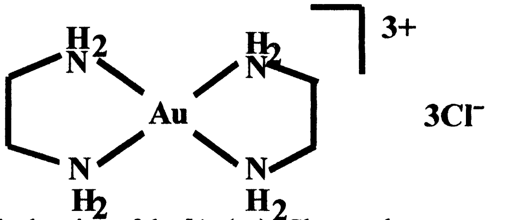

**Scheme I** Schematic drawing of the $[Au(en)_2]Cl_3$ complex

Since the $[Au(en)_2]Cl_3$ complex is water soluble and fairly stable under physiological conditions we investigated in more detail its interactions *in vitro* with DNA, the probable target for several antitumor metal complexes [13]. Given the fact that $[Au(en)_2]Cl_3$ is isoelectronic and isostructural with $[Pt(en)_2]Cl_2$, it is reasonable to hypothesize that its interaction with DNA may reproduce the main features of the $[Pt(en)_2]Cl_2$/DNA interaction. With this in mind we followed the reaction of $[Au(en)_2]Cl_3$ with calf thymus DNA through various spectroscopic techniques such as spectrophotometry, circular dichroism and analysis of DNA melting profiles; for comparison purposes similar experiments were carried out on the parent compound bisethylendiamine platinum(II) dichloride.

### MATERIALS AND METHODS

$[Au(en)_2]Cl$ was synthesized as reported in literature [12]. The purity of the compound was checked through elemental analysis and $^1H$ NMR spectroscopy. $[Pt(en)_2]Cl_2$ and *Calf thymus* DNA were purchased from Sigma Chemical Company. All other reagents used in this study were of analytical grade and were commercially available.
Electronic absorption spectra were measured with a Perkin Elmer Lambda 20 Bio instrument.

Electronic spectra of $[Au(en)_2]Cl$ $2x10^{-4}$ M were recorded before and after addition of *calf thymus* DNA (r= 0.2) in the phosphate 50 mM, NaCl 4 mM, pH 7.4 buffer.

All melting measurements were carried out in the $10^{-2}$ M $NaClO_4$ and $10^{-3}$ M NaCl buffer. *Calf thymus* DNA was dissolved in the buffer and DNA concentration determined by absorption measurements at 260 nm. DNA concentration was equal to $3.6x10^{-5}$ M [nucleotide]. DNA was treated with $[Au(en)_2]Cl_3$ or $[Pt(en)_2]Cl_2$ at mol/bp ratios r= 0.1, and each sample was incubated for 1 hour at room temperature.

Thermal denaturation experiments were performed in quartz cuvettes with a Perkin Elmer Lamba 20 Bio spectrophotometer equipped with a thermostated cell as described in reference. Samples were continuously heated with a rate of temperature increase of 0.5°C/min while monitoring the absorbance changes at 260 nm. The investigated interval of temperature ranged from 50°C to 90°C. Upon reaching 90°C, samples were cooled back to 40°C in order to follow the renaturation process. Values for melting temperatures ($T_m$) and for the melting interval ($\Delta T$) were determined according to the reported procedures [14].

CD spectra were recorded at increasing complex/*calf thymus* DNA ratio after mixing on a Jasco J600 dichrograph operating at room temperature, interfaced with a PC, and analyzed through the standard Jasco software package; either $[Au(en)_2]Cl_3$ or $[Pt(en)_2]Cl_2$ were added at the ratios r=0.1, r=0.2, r=0.5, r=1.0, r=2.0, r=4.0, r=10.0. DNA concentration (expressed in basepair) was $8.9x10^{-5}$M in buffer phosphate 50mM, NaCl 4 mM, pH=7.4. The CD technique is indeed very sensitive to even minor conformational changes of the DNA conformation produced by ligand binding [15].

### RESULTS AND DISCUSSION

The reaction of the bisethylenediamine gold(III) complex with calf thymus DNA was first analyzed through absorption spectroscopy. It is observed that addition of saturating amounts of calf thymus DNA to a buffered solution containing $[Au(en)_2]Cl_3$ does not alter the characteristic charge transfer bands of the $[Au(en)_2]Cl_3$ complex (Figure 1). This means that addition of calf thymus DNA does not produce any major perturbation of the gold(III) chromophore.

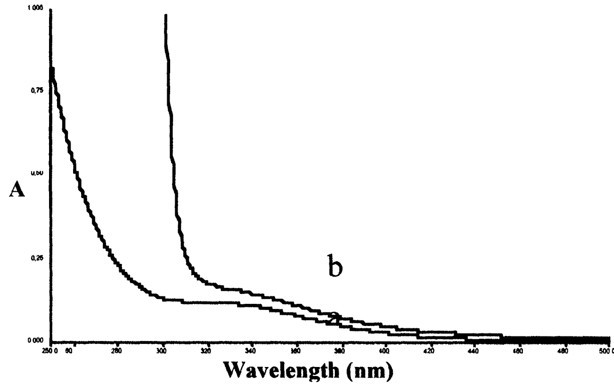

**Figure 1.** Absorption electronic spectra of $[Au(en)_2]Cl_3$ before (**a**) and after addition (**b**) of a saturating amount of calf thymus DNA.

To establish in more detail whether binding of $[Au(en)_2]Cl_3$ brings about any significant conformational change of the DNA double helix CD spectra of calf thymus DNA were carried out at increasing $[Au(en)_2]Cl_3$ to calf thymus DNA ratios. The profile of the CD titration of calf thymus DNA with $[Au(en)_2]Cl_3$ is shown in Figure 2.

Upon addition of small amounts of $[Au(en)_2]Cl_3$, minor changes of the B-type CD spectrum of calf thymus DNA are observed that reach completion at r= 0.1; for higher gold to DNA ratios more important changes of the CD spectrum are detected that are diagnostic of larger conformational changes. Remarkably addition of excess sodium cyanide causes complete reversal of the CD effects implying that the observed changes are a direct consequence of $[Au(en)_2]Cl_3$ binding and that they are reversible upon gold removal. For comparison purposes a CD titration of calf thymus DNA with $[Pt(en)_2]Cl_2$ was carried out under the same experimental conditions; the resulting spectral profiles are shown in Figure 3.

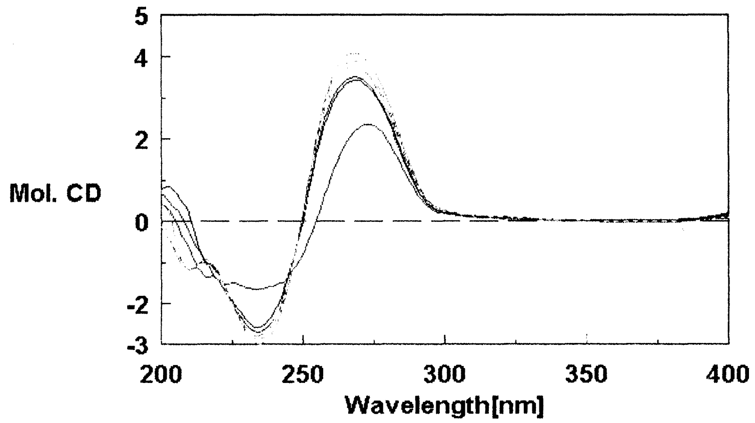

**Figure 2** CD titration of calf thymus DNA with increasing amounts of the bisethylendiamine gold(III) complexes. $[Au(en)_2]Cl_3$ was added at the following ratios: r=0.1, r=0.2, r=0.5, r=1.0, r=2.0, r=4.0, r=10.0 (from top to the bottom on the positive band at 260 nm and the reversal on the negative band at 240 nm).

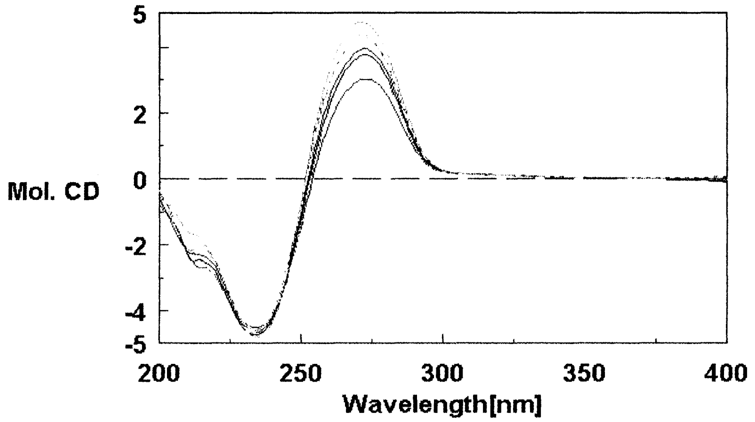

**Figure 3** CD titration of calf thymus DNA with increasing amounts of the bisethylendiamine platinum(II) complexes. $[Pt(en)_2]Cl_2$ was added at the following ratios: r=0.1, r=0.2, r=0.5, r=1.0, r=2.0, r=4.0, r=10.0 (from top to the bottom on the positive band at 260 nm and the reversal on the negative band at 240 nm).

From inspection of the spectra it is apparent that $[Pt(en)_2]Cl_2$ produces effects that are very similar, but not identical, to those produced by $[Au(en)_2]Cl$ . Again a biphasic behaviour is observed implying occurrence of both high affinity and low affinity binding of $[Pt(en)_2]Cl_2$ to DNA.

Finally, the effects of binding of both $[Au(en)_2]Cl_3$ and $[Pt(en)_2]Cl_2$ on the stability of DNA double helix were investigated through analysis of the helix-to-coil transition. It is found that addition of $[Au(en)_2]Cl_3$, at r=0.1, results into a modest increase of the melting temperature ($T_m$) with $\Delta T_m \cong 1.0°C$; a similar effect is produced by the addition of equimolar amounts of $[Pt(en)_2]Cl_2$ ($\Delta T_m \cong 0.6°C$). Again these data imply a similar mode of interaction.

### CONCLUSIONS

In conclusion the present results show that the cytotoxic complex $[Au(en)_2]Cl_3$, that is fairly stable under physiological conditions, binds calf thymus DNA non covalently with an affinity constant comparable to that of $[Pt(en)_2]Cl_2$. The interaction is electrostatic in nature and reversible, and brings about a modest conformational change and a slight stabilization of the double helix. Given the strict structural relationship of $[Au(en)_2]Cl_3$ to $[Pt(en)_2]Cl_2$ and the similar profile of the CD titrations it is suggested that $[Au(en)_2]Cl_3$ binds DNA in a similar fashion to $[Pt(en)_2]Cl_2$. According to the model proposed by Collins et al. such interaction should occur through minor groove binding of the cationic gold(III) complex, in correspondence of AT rich regions.

### REFERENCES

1. *Met. Ions Biol. Syst.* Sigel H. and Sigel A. editors, vol. 33, Marcel Dekker, New York, **1996**;
2. *Bioinorganic Chemistry*, (Bertini, I., Gray, H.B., Lippard S.J. and Valentine J.S. editors) University Science Books, Sausalito, California, **1994**;
3. Xu, Q., Shoemaker, R.K., Braulin, W.H. *Biophys. J.* **1993**, 65, 1039;
4. Xu, Q., Jampani, S.R.B. and Braulin, W.H. *Biochemistry* **1993**, 32, 11754;
5. Xu, Q., Jampani, S.R.B. Deng, H. and Braulin, W.H. *Biochemistry* **1995**, 34, 14059;
6. David, S.S., Barton, J.K. *J. Am. Chem. Soc.* **1993**, 115, 2984;
7. Terbrueggen, R.H., Barton J.K. *Biochemistry* **1995**, 34, 14059;
8. Hudson, B.P., Dupureur, C.M., Barton J.K. *J. Am. Chem. Soc.* **1995**, 117, 9379;
9. Franklin, C.A., Fry, J.V. and Collins, J.G. *Inorg. Chem.* **1996**, 35, 7541-7545;
10. Calamai, P., Carotti, S., Guerri, A., Mazzei, T., Messori, L., Mini, E., Orioli, P. and Speroni, G. P. *Anticancer Drug Des.* **1998**, 13, 67-80;
11. Calamai, P., Carotti, S., Guerri, A., Messori, L., Mini, E., Orioli, P. and Speroni, G.P. *J. Inorg. Biochem.* **1997**, 66, 103-109;
12. Abbate, F., Carotti, S., Guerri, A., Marcon G., Mazzei, T., Messori, L., Mini, E., Orioli, P. and Zanello, P., *J. Med. Chem.* **2000**, 43 (19), 3541-3548;
13. *Metal Complexes in Cancer Chemotherapy* (Keppler, B. K. ed.), p.37, VCH: Weinheim, **1992;**
14. Haroutiunian, S.G., Bruni, B., Monnanni, R., Orioli, P., Mangani, S. *Inorg. Chim.Acta* **1992**, 184, 127-131;
15. Gray, D.M., Ratliff, R.L., Vaughan, M.R. *Methods Enzyimol.* **1992**, 211, 389.

**Received: December 8, 2000 - Accepted: December 14, 2000 - Received in revised camera-ready format: December 15, 2000**

